# Supplementary figures and images for: The genomic landscape of metastasis in treatment-naïve breast cancer models
Source: PLoS Genet. 2020 May 28;16(5):e1008743. doi: 10.1371/journal.pgen.1008743 (PMC7282675; doi:10.1371/journal.pgen.1008743)

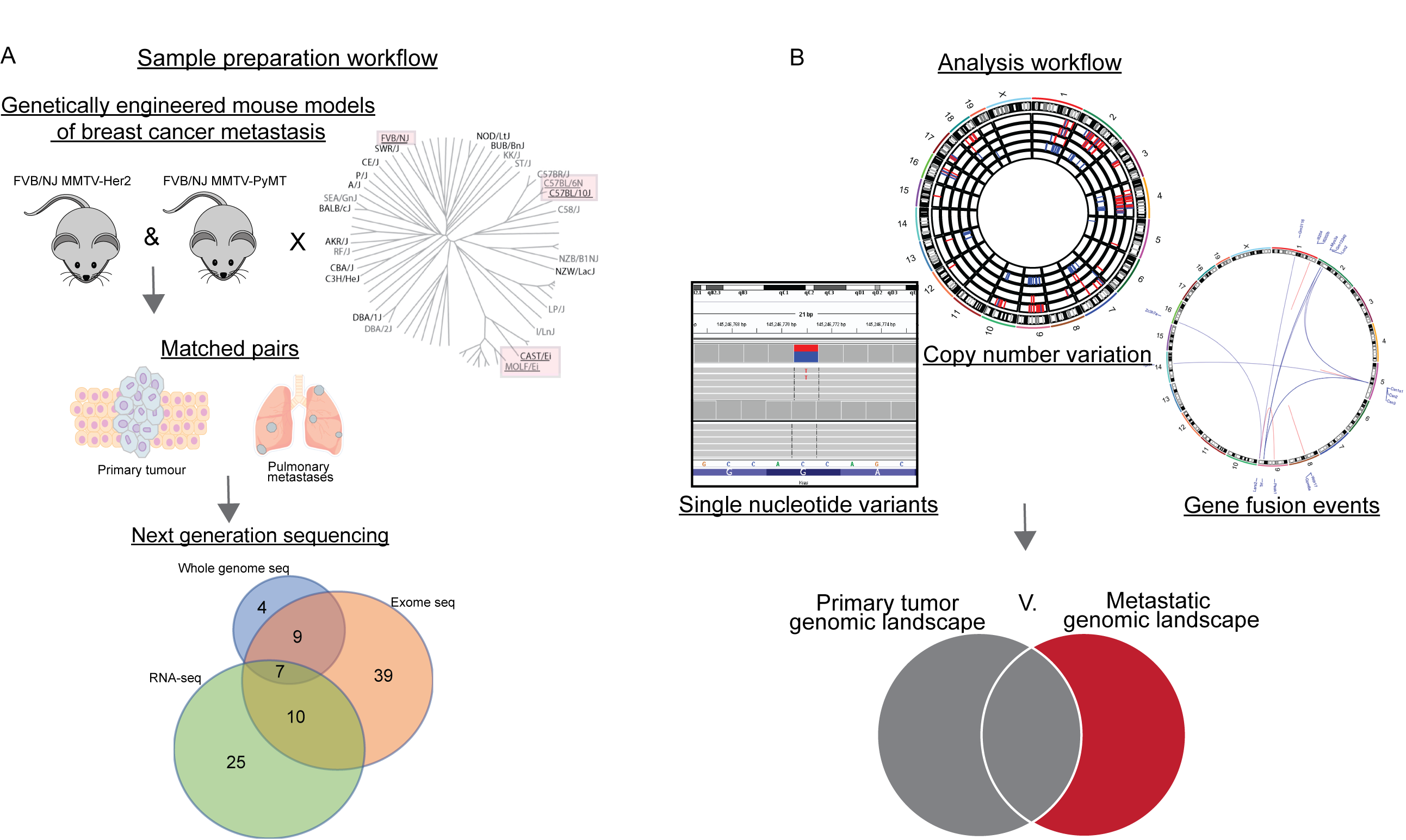

Supplement: S1 Fig — A. Schematic of sample collection for analysis. Preclinical mouse model development followed by sample collection from 14 FVB x MMTV-Her2 and 80 outcrossed MMTV-PyMT mice. Overlapping analyses performed on paired samples, numbers in Venn diagram represent number of animals. B. Schematic of analysis workflow. Next generation sequence data was analyzed for single nucleotide variants, copy number variants, and structural variants. The results were then filtered for those events enriched in metastatic tissue only. (TIF) [file pgen.1008743.s001.tif]

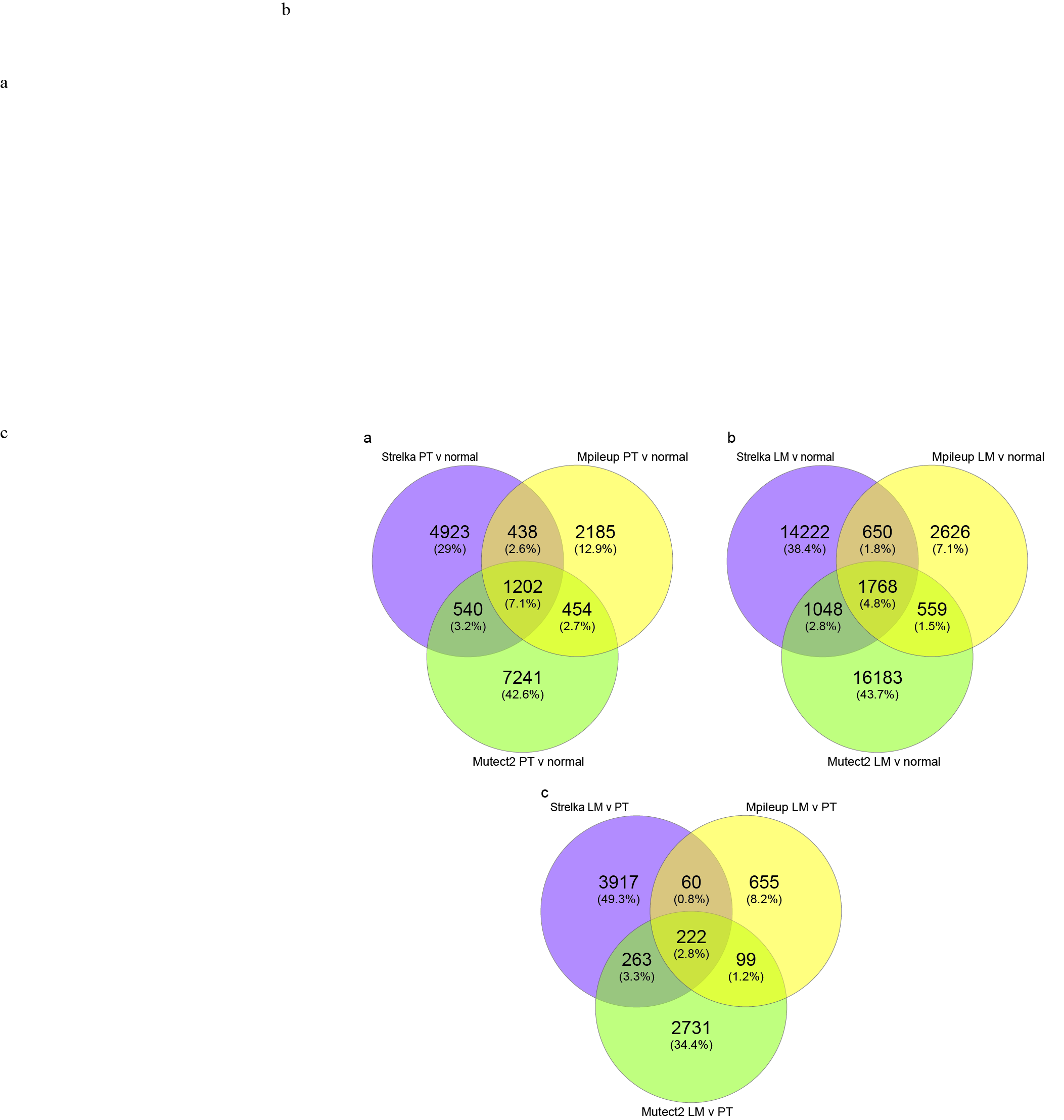

Supplement: S2 Fig — Overlap of three SNV calling algorithms, Strelka (purple), Mpileup (yellow), and Mutect2 (green), used with exome-seq data collected from primary tumor (PT) and lung metastases (LM) from 65 mice. A. SNVs called in PT tissue when compared to normal (strain-specific) gDNA. B. SNVs called in LM when compared to normal (strain-specific) gDNA. c. SNVs called in LM when compared to paired PT tissue using 0.3 allele frequency cutoff. (TIF) [file pgen.1008743.s002.tif]

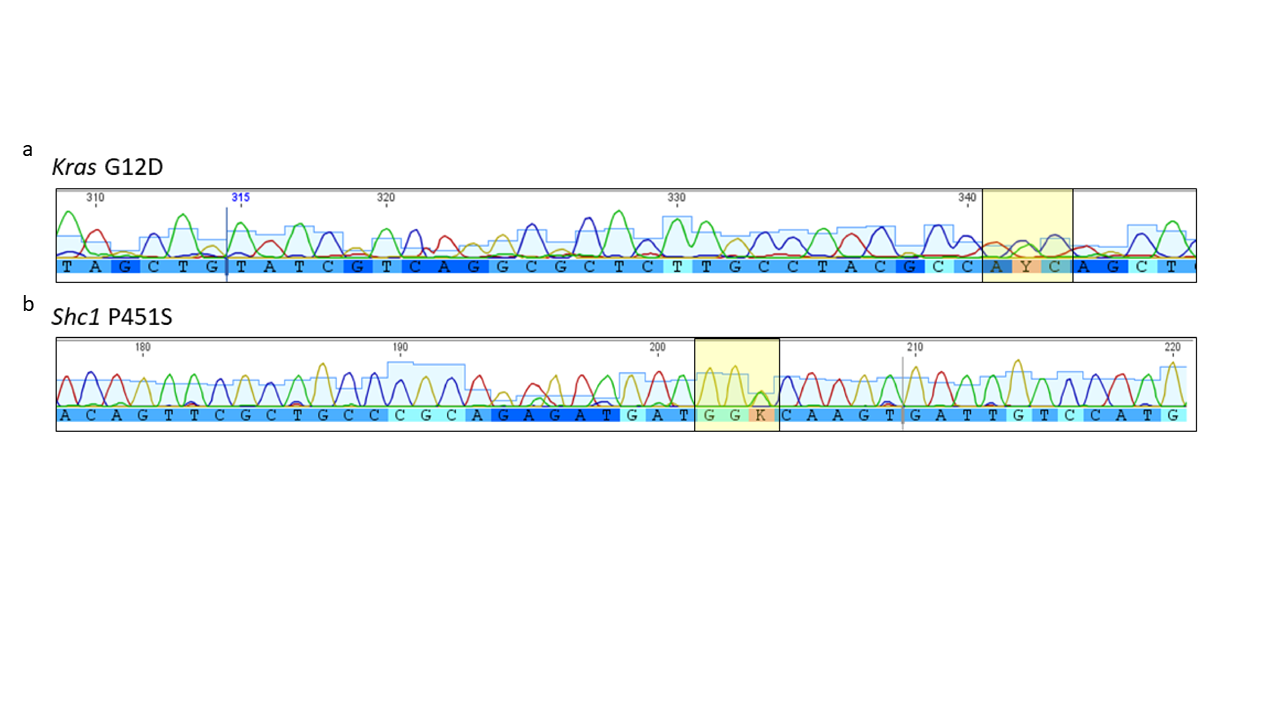

Supplement: S3 Fig — A. C (blue trace)-T (green trace) SNV within the Kras gene resulting in the G12D amino acid substitution, Y indicates ambiguity in calling T or C. B. G (yellow trace)-T (green trace) substitution within the Shc1 gene resulting in the P561S amino acid substitution. K indicates ambiguity in calling T or G. (TIF) [file pgen.1008743.s003.tif]

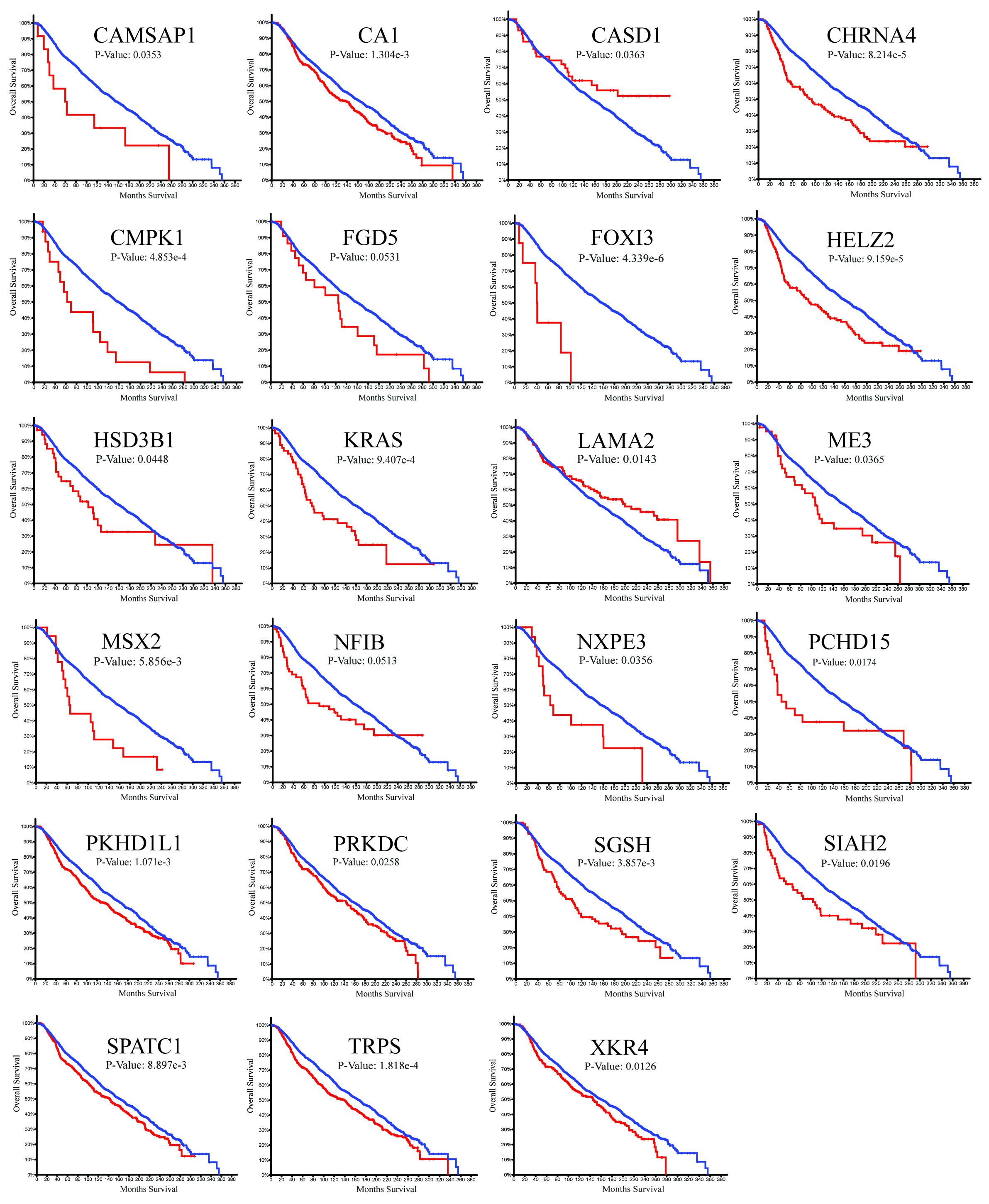

Supplement: S4 Fig — Kaplan-Meier plots generated using METABRIC for 23 genes identified with metastasis-driver SNVs by exome-seq in mice that significantly stratify patient survival when altered in primary tumor tissue (blue = no CNV, red = CNV present). (TIF) [file pgen.1008743.s004.tif]

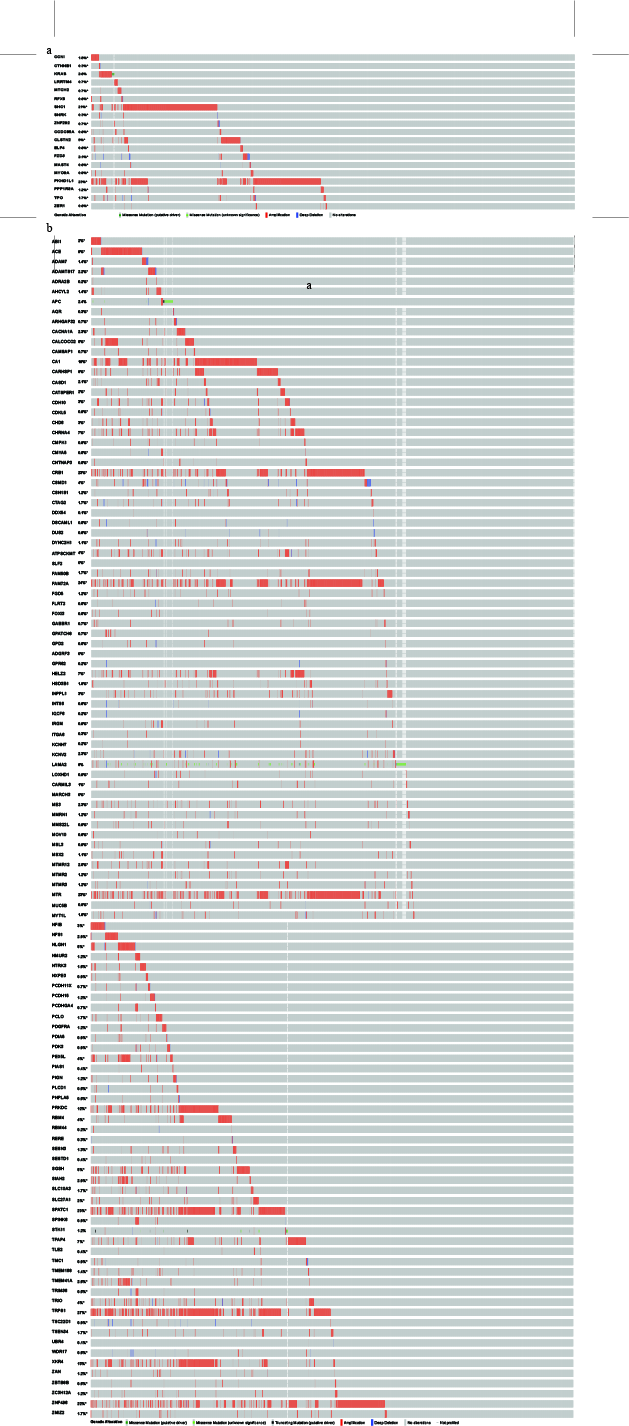

Supplement: S5 Fig — Oncoprint schema from the METABRIC human primary tumor dataset showing copy number variation rates of the A. 17 genes with recurrent SNVs and B. 147 singly mutated genes identified by exome-seq as putative metastasis-driver mutations (red = amplification, blue = deletion, green = SNV). (TIF) [file pgen.1008743.s005.tif]

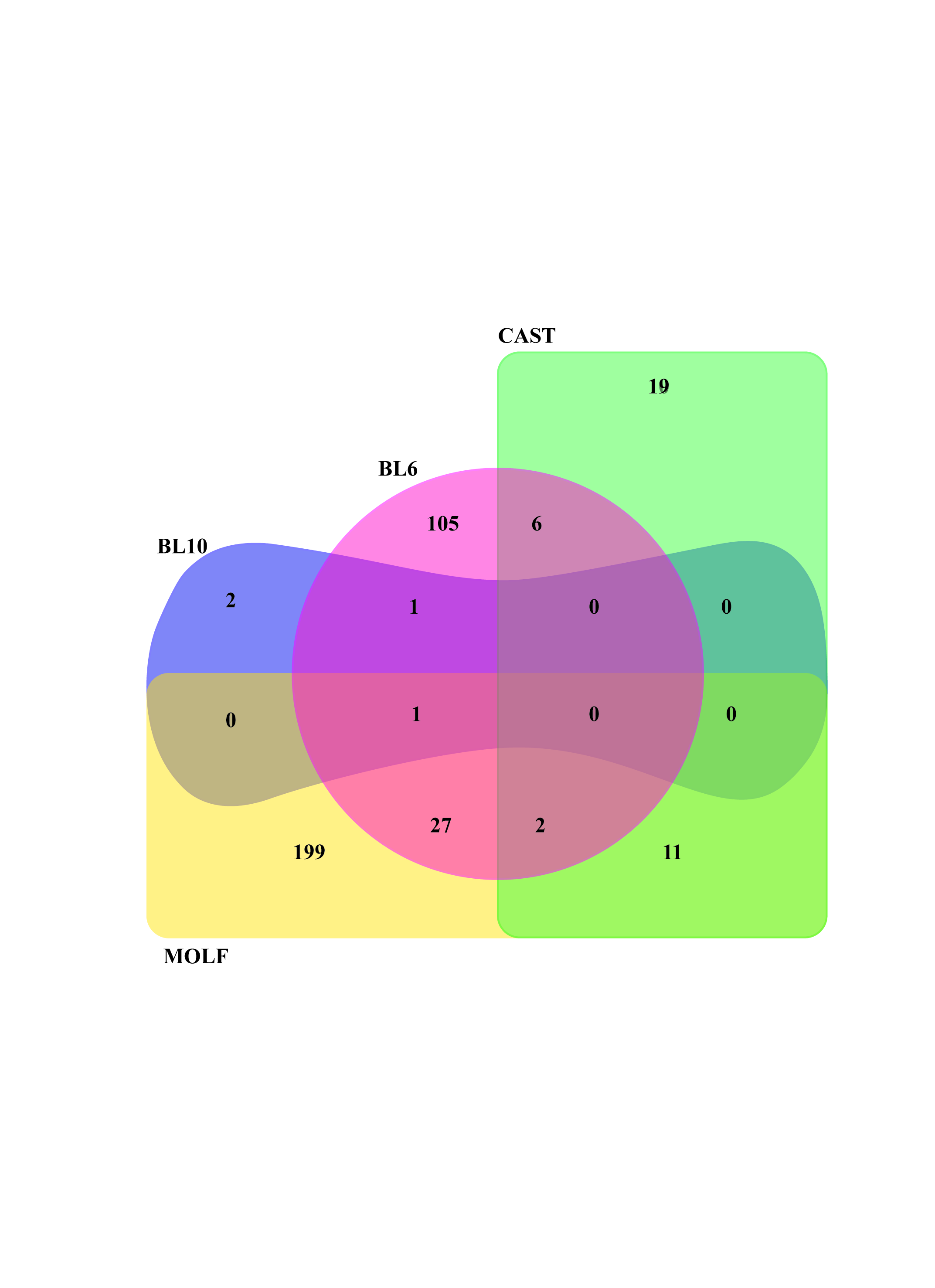

Supplement: S6 Fig — Numbers represent the number of genes, and numbers in overlapping regions represent the number of common CNV-associated CNVs. (TIF) [file pgen.1008743.s006.tif]

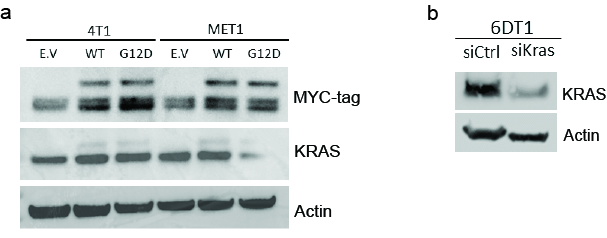

Supplement: S7 Fig — A. Western blot showing expression of MYC-tagged KRAS and total KRAS in 4T1 and MET1 transduced with empty vector (EV), Kras wildtype (WT), and Kras G12D (G12D). B. Western blot showing knock down of KRAS in 6DT1 cells 24 hours after transfection siCtrl or siKras. (TIF) [file pgen.1008743.s007.tif]

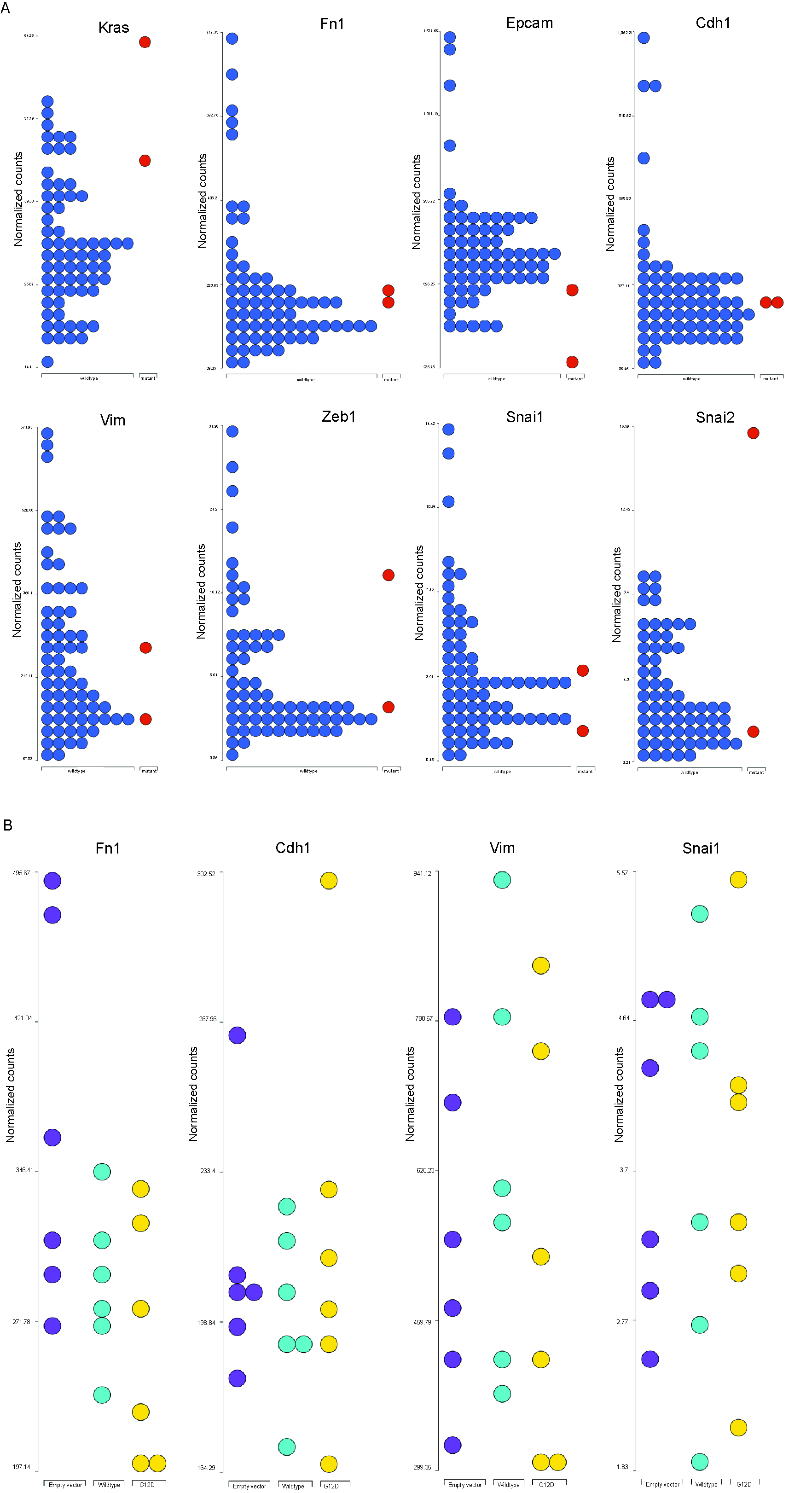

Supplement: S8 Fig — A. Dot plots showing normalized Kras and EMT gene transcript counts by RNA-seq of metastatic nodules from PyMT and Her2 animals with Kras wildtype (blue) or Kras mutations (red). B. Dot plots showing normalized EMT gene transcript counts by RNA-seq of 4T1 cells stably transduced with empty vector (purple), Kras wildtype (blue), or Kras G12D (yellow) expression vectors. 4. (TIF) [file pgen.1008743.s008.tif]
